# Optimal protocol for in vitro polyploid induction of *Cymbidium aloifolium* (L.) Sw

**DOI:** 10.1186/s12870-023-04314-8

**Published:** 2023-06-02

**Authors:** Worasitikulya Taratima, Khoirista Noor Rohmah, Kongtong Plaikhuntod, Pitakpong Maneerattanarungroj, Attachai Trunjaruen

**Affiliations:** 1 Department of Biology, Faculty of Science, Khon Kaen University, Khon Kaen, 40002 Thailand; 2 Salt Tolerant Rice Research Group, Khon Kaen University, Khon Kaen, 40002 Thailand; 3 Faculty of Science, Khon Kaen University, Khon Kaen, Thailand; 4 Faculty of Veterinary Medicine, Khon Kaen University, Khon Kaen, 40002 Thailand

**Keywords:** Chromosome number, Colchicine, *Cymbidium*, Leaf anatomy, Orchid, Tetraploid

## Abstract

**Background:**

*Cymbidium aloifolium* is a popular ornamental flower in Thailand with both economic and medical values. Polyploid induction techniques are used to improve plant quality. This study identified polyploidy levels of *C. aloifolium* induction by colchicine. Protocorms of *C. aloifolium* were treated on solid New Dogashima Medium (NDM) with various concentrations of colchicine (0, 0.01, 0.02, 0.03, 0.04 and 0.05% w/v) for 2, 4 and 8 weeks.

**Results:**

Colchicine effectively induced plant polyploidy. Tetraploid plants were found after treatment with 0.03% and 0.04% colchicine for 8 weeks, while at increased concentration and duration, survival, response and growth performance decreased. Tetraploid plants showed the lowest growth performance but highest size of stomatal and epidermal cells. Growth performance and leaf surface anatomy data were analyzed by Pearson’s correlation and PCA. Results showed that stomatal and epidermal cell sizes had strongly negative correlations with other variables, while HCA revealed that stomatal and epidermal cell sizes of tetraploid plantlets were larger but stomatal and epidermal cell densities decreased when compared with the diploids.

**Conclusion:**

Colchicine at suitable concentrations and duration produced polyploid plants with alteration of morphological and anatomical traits. This study provides potential information to support orchid quality production as ornamental plants and a source of pharmaceutical raw materials.

## Background

Orchids are widely cultivated as ornamental blooming crops with many benefits, especially in the field of floriculture. Orchids are also used for medicinal and cultural purposes in many global cultures [1]. Genera of economically important orchids include *Cymbidium*, *Dendrobium*, *Spathoglotis* and *Vanda*. Thailand has tremendous orchid biodiversity and is well-known for exporting both cut flowers and potted orchids [2]. *Cymbidium aloifolium* is a popular orchid found in Thailand with economic value [3, 4]. Moreover, *Cymbidium* is also used in traditional medicine to relieve inflammation because of its rich phytochemical contents [5]. Nowadays, orchid habitats are in decline and plant populations need to be improved [3]. Anthropogenic environmental damage is threatening the abundance of orchid populations, while demand for the plants is increasing. To overcome this problem, efforts to increase the number of good quality orchids have concentrated on genetic enhancement through mutation [6].

Polyploidy, as the heritable condition of possessing more than two complete sets of chromosomes, is an effective method to produce better crop quality [7]. Alteration of chromosome numbers also results in changes in the morphological characteristic of plants. Polyploid induction can be used to produce larger flowers with more intense color and greater endurance [8]. Polyploid plant induction can be performed by applying colchicine as a chemical polyploid inducer that causes chromosome duplication followed by cell size enlargement. Microtubules are desirable targets in colchicine application because they match with tubulin protein, resulting in inhibition of spindle fiber formation during anaphase [9]. This causes the depolymerization of microtubules because colchicine attaches to the ends of microtubulin polymers and the subsequent subunits cannot join to form spindle fibers [10]. At anaphase, chromatic fibers cannot attach to the polar cells, so the chromosomes increase from 2 × to 4 × as polyploids [9]. Colchicine treatment can be conducted in two medium states. The first involves shaking the protocorm-like bodies (PLBs) in colchicine liquid medium, while the second involves treatment in colchicine solid medium. Polyploid induction is an effective technique to improve orchid development. However, how different colchicine concentrations affect orchid polyploidy levels must first be determined to maximize genetic enhancement and improvement.

## Results

**Fig.1:**
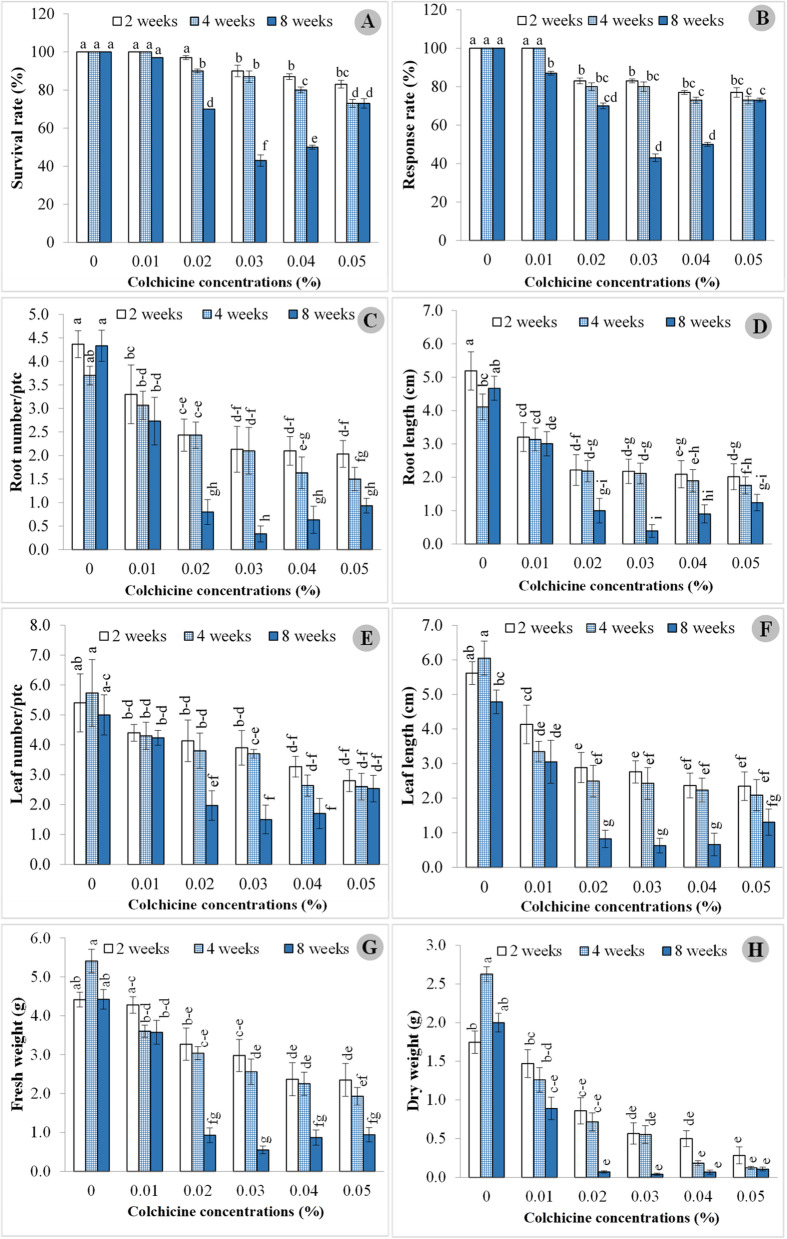
Growth performances of *C. aloifolium* after culture in solid colchicine medium at different concentrations and durations: **A** survival rate; **B** response rate; **C** root number; **D** root length; **E** leaf number; **F** leaf length; **G** fresh weight and **H** dry weight. Data are shown as mean ± SE

Colchicine effectively induced plant polyploidy in certain treatments, with 100% survival and response observed in protocorms treated with 0% and 0.01% colchicine for 2 weeks, and 0.01% colchicine for 4 weeks. Survival percentages decreased for other concentrations and durations (Fig. 1A). Polyploid plants were identified as tetraploid in the 0.03% and 0.04% colchicine treatments at 8 week durations, exhibiting the lowest survival percentage values of 43% and 50%, respectively, compared to the other treatments. In correlation with these findings, the response rate showed improvement, with the highest percentage (100%) observed in treatments using 0% colchicine and 0.01% colchicine for both 2 and 4 weeks (Fig. 1B). Conversely, the lowest response was observed in polyploid plants treated with 0.03% colchicine (43% response) and 0.04% colchicine (50% response).

**Fig. 2:**
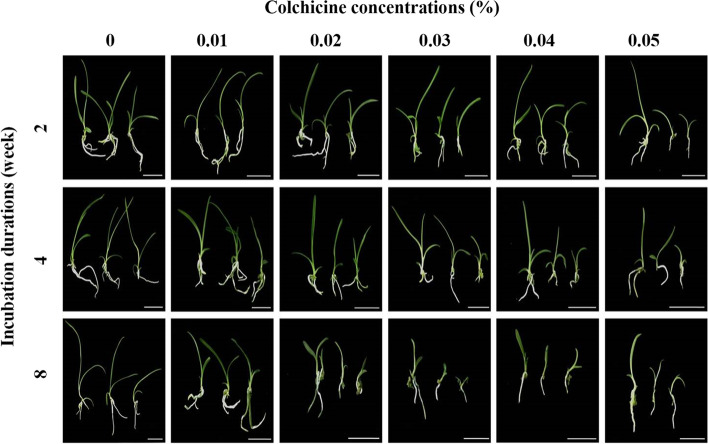
Plantlets of *C. aloifolium* after culture in solid colchicine medium at different concentrations and durations (scale = 3 cm)

Growth performance of *C. aloifolium* was calculated by measuring root, leaf and weight. Highest root and leaf growth were recorded in the control group (0% colchicine), while protocorms in medium containing colchicine had lower growth rates, especially at 0.03% and 0.04% colchicine for 8 weeks. In the control medium (0% colchicine), highest growth was recorded at 2 weeks of colchicine exposure with root number 4.37 and length 5.19 cm (Fig. 1C and D). Good results were obtained after 8 weeks of colchicine treatment. The medium treated with 0% colchicine for 4 weeks produced the highest leaf number at 5.73 and also the longest leaves at 6.05 cm (Fig. 1E and F). Furthermore, fresh and dry weights were highest at 5.40 g and 2.63 g, respectively were observed after 4 weeks of treatment with 0% colchicine (Fig. 1G and H). The lowest growth of root, shoot and leaf were found in treatments with 0.03% and 0.04% colchicine at 8 weeks. Treatment at 0.03% gave lower size than 0.04%. Root number in the 0.03% treatment was 0.33 with root length 0.39 cm, while 0.04% colchicine gave 0.63 roots and length at 0.90 cm. Leaf number at 1.50 and length at 0.63 cm were yielded from the 0.03% treatment, while higher leaf number and leaf length were attained at 0.04% as 1.70 and 0.66 cm, respectively. Plant weights at 0.55 g fresh and 0.04 g dry weight were obtained in 0.03% colchicine, with higher weights from 0.04% colchicine at 0.87 g and 0.07, for fresh and dry leaves, respectively. Growth performances of *C. aloifolium* plantlets treated with colchicine are shown in Fig. 2.

**Fig. 3:**
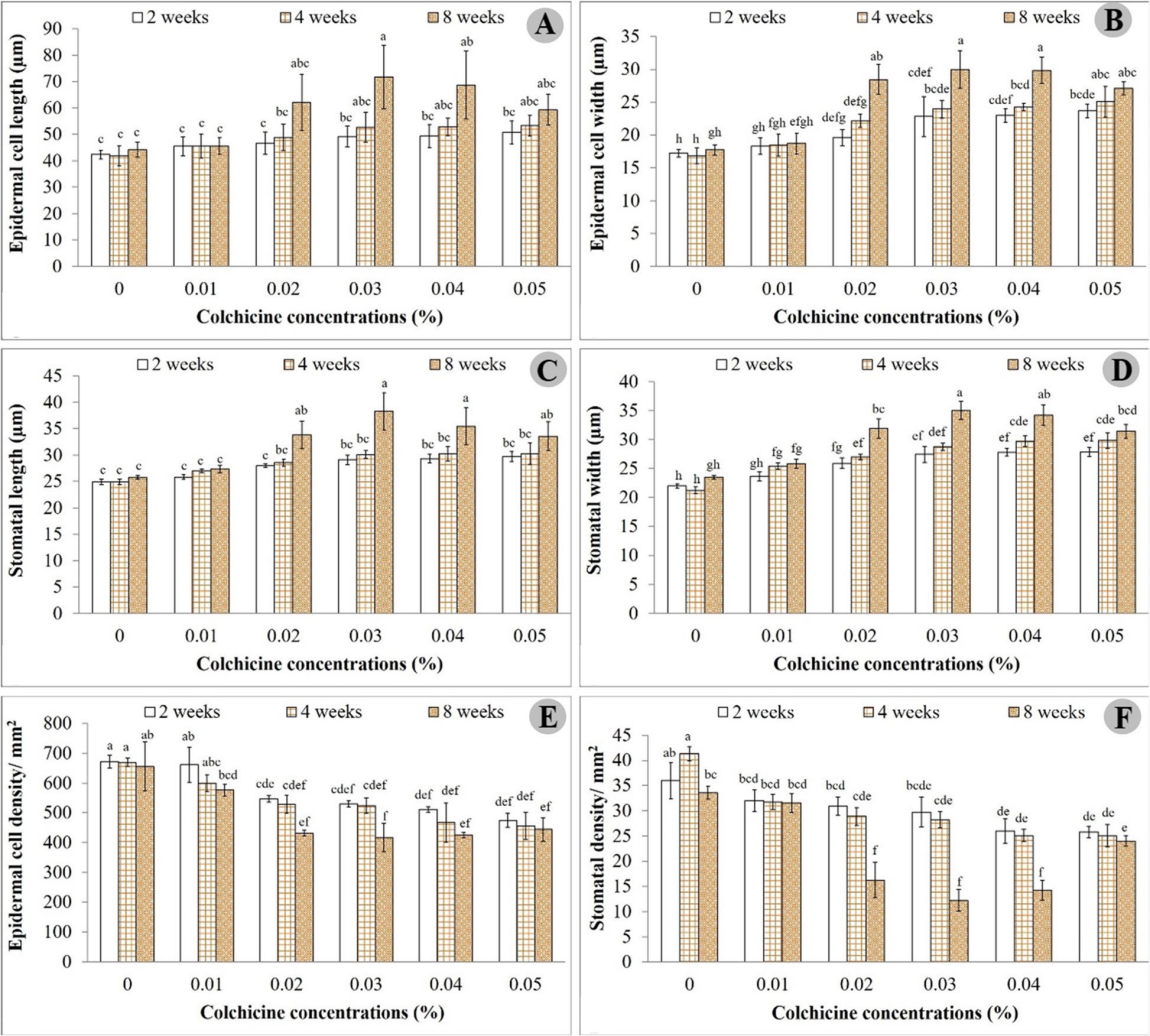
Epidermal cell size and stomatal size of *C. aloifolium* after culture in solid colchicine medium at different concentrations and durations: **A** epidermal cell length; **B** epidermal cell width; **C** stomatal length; **D** stomatal width; **E** epidermal cell density and **F** stomatal density. Data are shown as mean ± SE

**Fig. 4:**
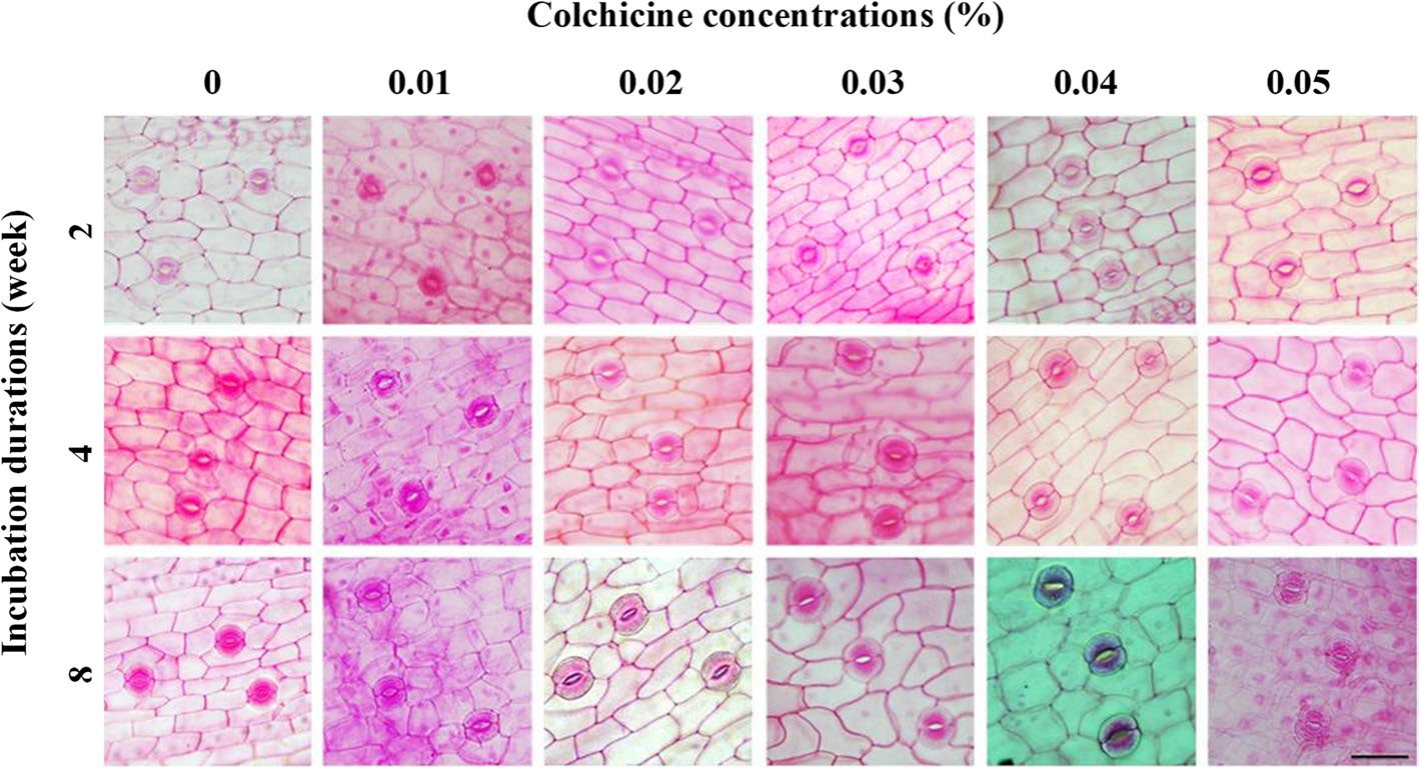
Epidermis and stomata of *C. aloifolium* after culture in solid colchicine medium at different concentrations and durations (scale = 50 µm)

Epidermal cell size and stomatal size were also recorded to observe the impact of colchicine. Media supplemented with 0.03% and 0.04% colchicine for 8 weeks duration showed significantly different epidermal cell size and stomatal size compared to other treatments. Specifically, 0.03% colchicine resulted in larger cell and stomatal sizes compared to 0.04% colchicine. The longest epidermal cell measured 71.72 µm followed by 68.63 µm, while the widest epidermal cell measured 29.98 µm followed by 29.85 µm in 0.03% and 0.04% colchicine treatment, respectively (Fig. 3A and B). Meanwhile, 0% colchicine generated smaller stomatal size than the other treatments, with shortest length 41.91 µm and width 16.84 µm. However, wider epidermal cells were produced in lower numbers. The lowest density at 416.89/mm^2^ was found at 0.03%, with 425.11/mm^2^ at 0.04% colchicine, while the highest density was attained in the untreated colchicine medium (Fig. 3E). In agreement with epidermal cells, 0.03% colchicine yielded larger stomata (38.30 µm length and 35.03 µm width) than 0.04% colchicine (35.50 µm length and 34.24 µm width) (Fig. 3C and D). Contrarily, stomatal density achieved minimum value in these two treatments, with the maximum value found at 0% colchicine. Highest density was 41.33/mm^2^ and lowest 12.22/mm^2^, followed by 14.22/mm^2^ (Fig. 3F). Stomatal sizes for all treatments are shown in Fig. 4.

**Fig. 5:**
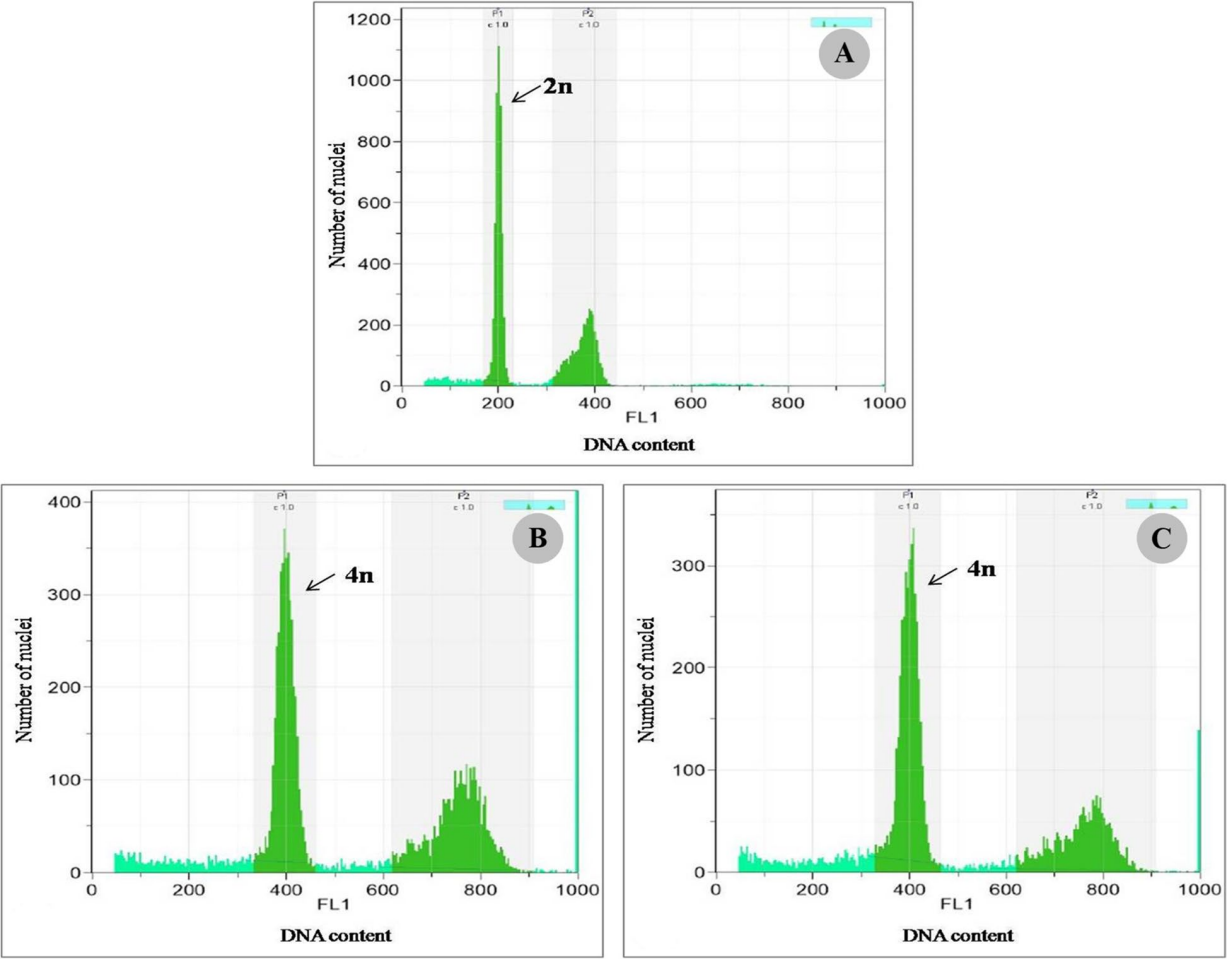
Flow cytometry of *C. aloifolium* after culture in solid colchicine medium at different concentrations: **A** control group (0% colchicine) (diploid); **B** 0.03% colchicine treatment for 8 weeks (tetraploid) and (**C**) 0.04% colchicine treatment for 8 weeks (tetraploid)

**Fig. 6:**
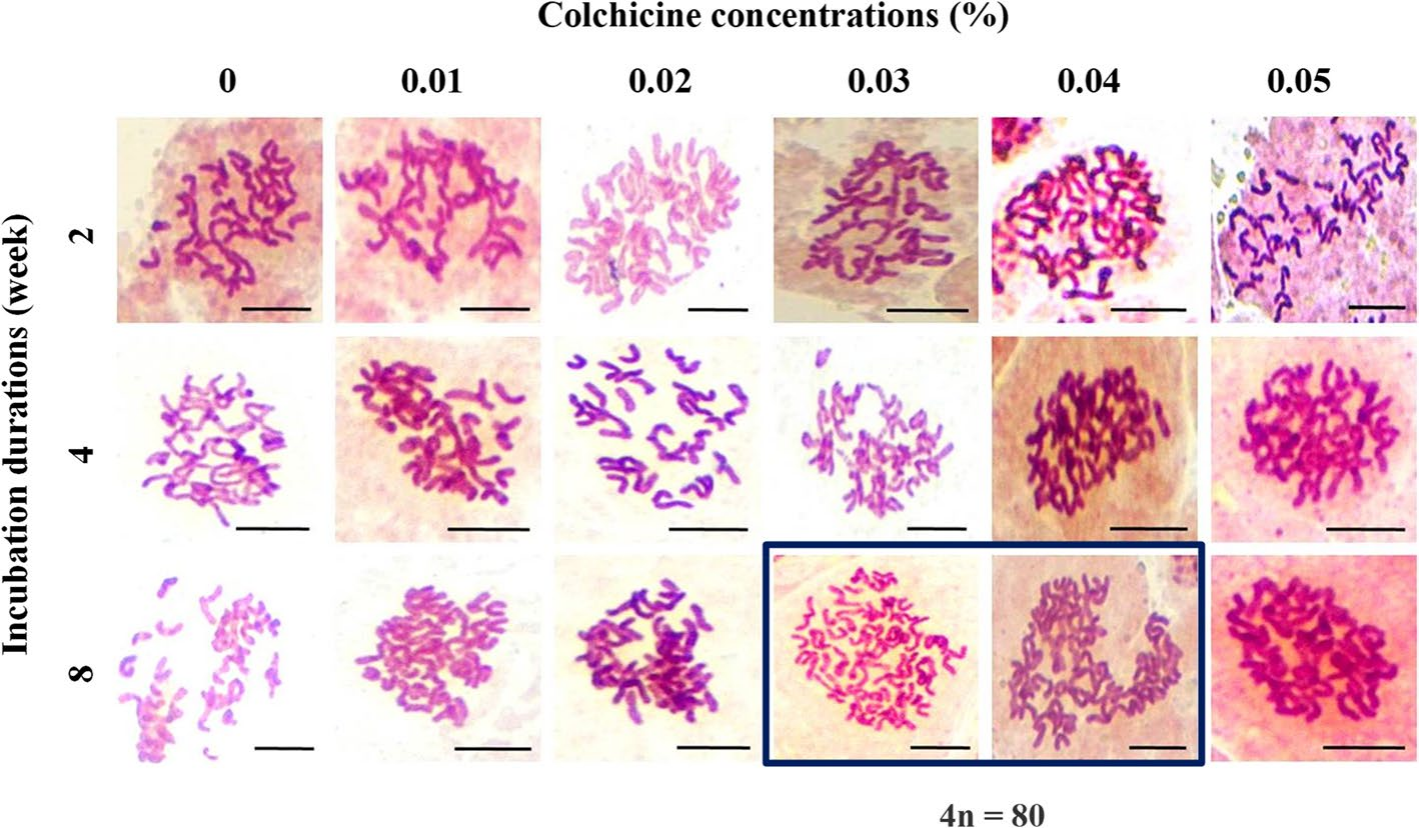
Chromosome number from root tip cell of *C. aloifolium* after culture in solid colchicine medium at different concentrations and durations. Treatments with 0.03% and 0.04% colchicine for 8 weeks incubation duration were tetraploid (4*n* = 80), while all other treatments were diploid (2*n* = 40) (scale = 10 µm)

Ploidy levels were analyzed using flow cytometry to detect polyploidy and showed plant DNA contents as a peak. Results indicated that treatments with 0.03% and 0.04% colchicine at 8-weeks duration gave tetraploid plants. Peak DNA content of the diploid was 400 (Fig. 5A), while the tetraploid was 800 with twice the DNA content of the other treatments (Fig. 5B and C). These results concurred with the chromosome counting technique that indicated colchicine treatment at 0.03% and 0.04% for 8 weeks had different numbers from the others. These two treatments had 80 chromosomes as tetraploid (4n), while the other treatments had 40 chromosomes as diploid (2n) (Fig. 6). This result indicated that colchicine in solid medium with long duration (in weeks) was appropriate to induce tetraploid plants in *C. aloifolium*. Furthermore, tetraploid plants generated lowest growth performance but produced the highest epidermal cell size and stomatal size.

**Fig. 7:**
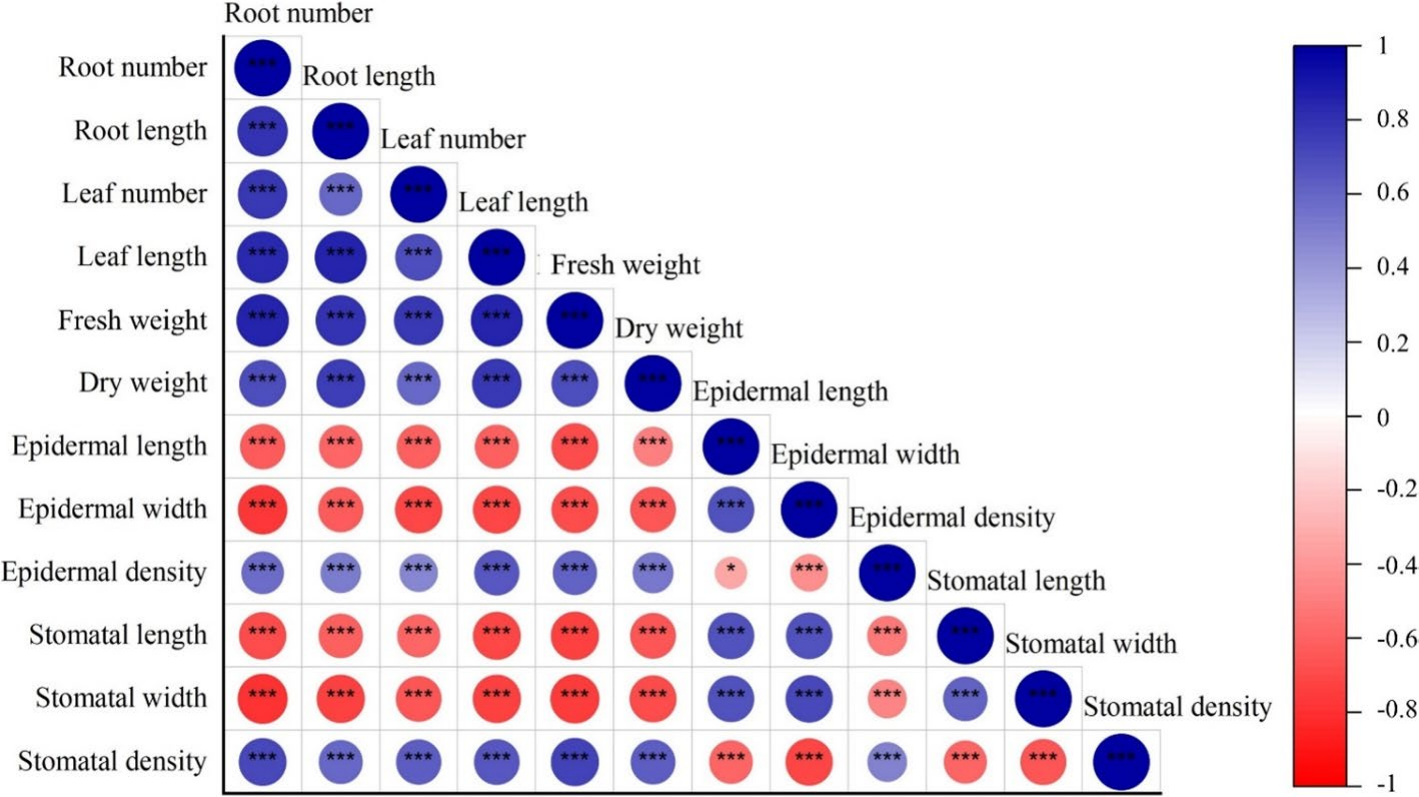
Pearson’s correlation between growth performance and leaf surface anatomical variables in response to different concentrations of colchicine and culture periods (*, *p* < 0.05; **, *p* < 0.01; ***, *p* < 0.001)

Pearson’s correlation was applied to analyze the relationships between growth performance and anatomical characteristics of the leaf surface. The relationships were also illustrated by a biplot using principal component analysis (PCA). Pearson’s correlation results exhibited strong negative and positive correlations between all the variables (Fig. 7). All growth performance variables and the densities of epidermal cells and stomata showed significant correlations with each other, while size of epidermal cells highly correlated with stomatal size (*p* < 0.001). Negative correlations were found between size of epidermal cells and stomata and their densities on the leaf surface. Similar negative correlations in sizes and all growth performance variables were also observed (*p* < 0.001).

PCA was used to visualize the relationships between the variables and to cluster.

**Fig. 8:**
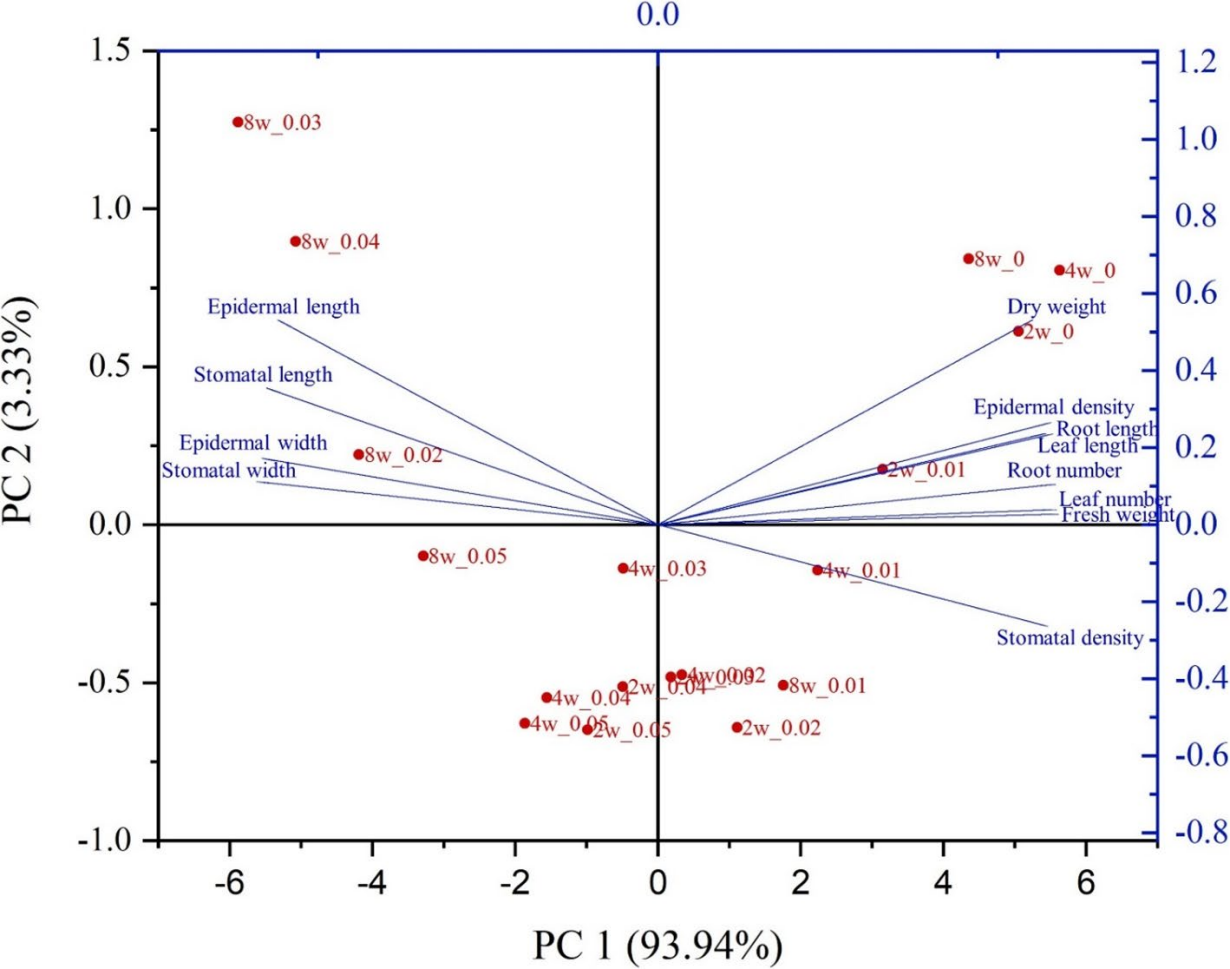
PCA biplot from PC1 and PC2 showing the relationships between growth performance and leaf surface anatomical variables, and distributions of diploid and tetraploid scores from all treatments

*C. aloifolium* depending on plantlet response. A biplot explaining 97.27% of the variance was created from PC1 and PC2 explaining 93.94% and 3.33% of the data variance, respectively. Dry weight, epidermal cell length and stomatal length were included in PC2, while other variables were included in PC1. Scores from all the treatments were plotted as a biplot. Results showed that diploid scores were distributed in all quadrants, while scores from tetraploid treatments and plant cultures with 0.03 and 0.04% colchicine for eight weeks were clustered in the upper left quadrant (Fig. 8).

**Fig. 9:**
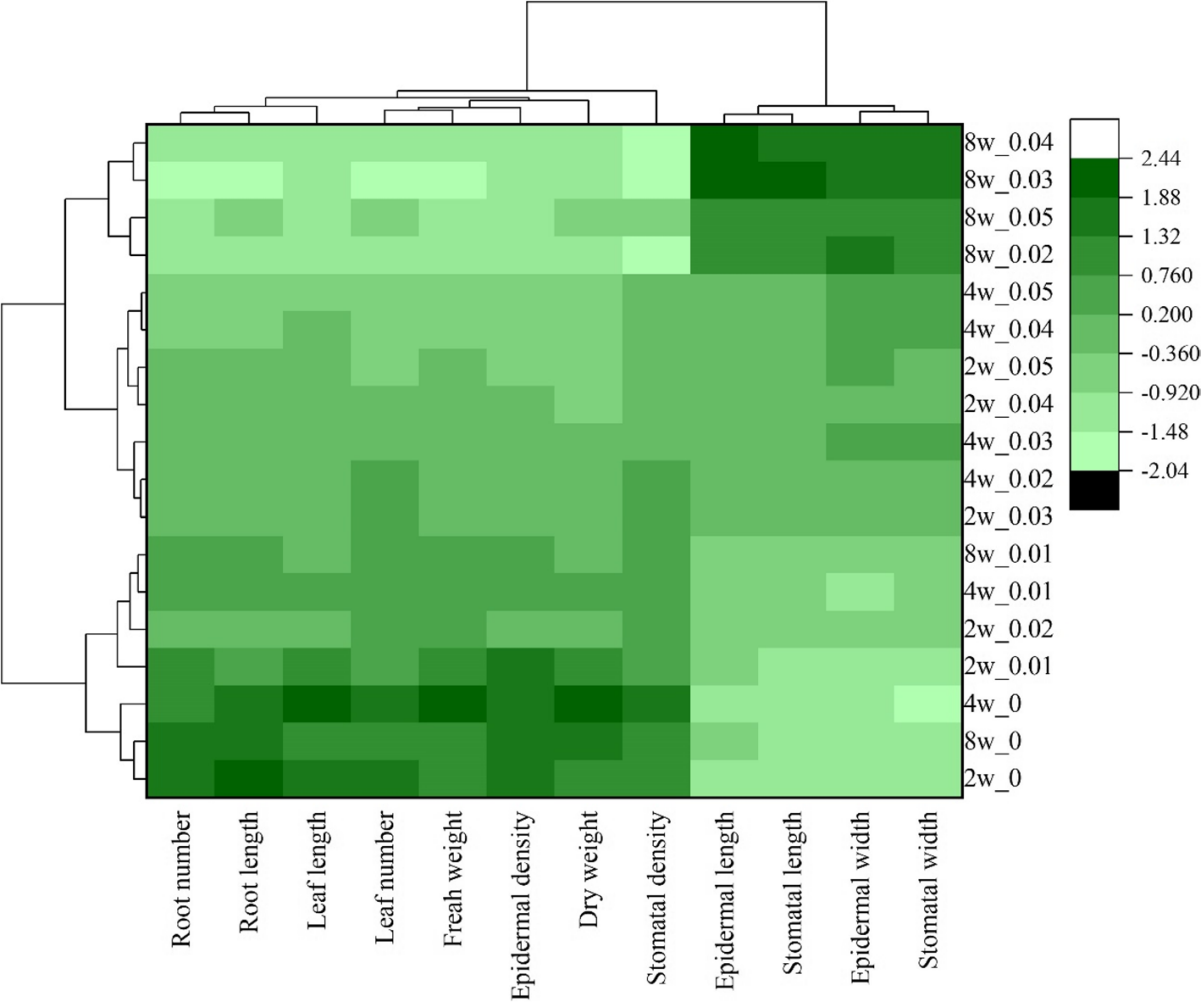
A heatmap and hierarchical cluster analysis showing the response patterns of *C. aloifolium* plantlets to culture on media containing different concentrations of colchicine for two, four and eight weeks

Similar data were used to further investigate the response of *C. aloifolium* plantlets to culture at different concentrations and time periods using a heatmap and HCA. Results identified variables as two clusters of growth performance and leaf surface anatomy. Cluster I consisted of all growth performance variables and epidermal and stomatal cell densities with stomatal and epidermal cell sizes in cluster II. The response patterns of *C. aloifolium* plantlets were divided into five major clusters depending on the same response pattern of the variables. Cluster I contained plantlets cultured on MS medium without colchicine for two to eight weeks, while plantlets undergoing treatments of 0.01% colchicine for two to eight weeks and 0.02% colchicine for two weeks were grouped in cluster II. Plantlets cultured on elevated colchicine concentrations (0.02 – 0.05%) for two and four weeks were grouped in cluster III, while plantlets cultured for eight weeks at 0.02% and 0.05% colchicine were grouped in cluster IV. Tetraploid *C. aloifolium* plantlets were obtained from the treatments of 0.03% and 0.04% colchicine cultured for eight weeks (Fig. 9).

## Discussion

Polyploid plants contain three or more chromosome sets in their cells. Colchicine is widely used as a chromosome doubling agent through in vitro propagation. Addition of colchicine impacted survival and response rate, growth performance, epidermal and stomatal cell sizes. Furthermore, polyploid analysis using flow cytometry and chromosome counting showed that colchicine effectively induced tetraploidy at 0.03% and 0.04% colchicine after 8 weeks. In diploid plants, the peak showed DNA content at 400 (Fig. 5A), while in these two treatments, DNA contents were 800 (Fig. 5B and C). Diploid plants showed two peaks in the histogram as the main peak of DNA content at 400 and another peak at more than 400. In this condition, the second peak suggested that some cells were still involved in the cell division process and not considered a polyploid plant. Treatments of colchicine with appropriate dose and duration resulted in polyploid plants by inhibiting the mitotic process in certain phases, which later supported chromosome doubling. As an anti-mitotic agent, colchicine inhibited cell division at the metaphase stage by disrupting spindle thread activities [11]. Colchicine easily binds with tubulin dimers that play a role in microtubule formation. This process prevented new dimers from attaching to the microtubule assembly side. As a result, disassembly and depolymerization of the microtubules occurred. Furthermore, they explained that as a consequence of the failed microtubule arrangement, the spindle threads did not form at the metaphase stage and the chromosomes were unable to separate into each pole and perform chromosome doubling.

Survival and response percentages decreased at higher colchicine concentrations and durations. The lowest rates were recorded in tetraploid treatments. This outcome concurred with previous reports that high concentrations and incubation durations impacted the survival percentages of *Dendrobium scabrilingue* [12] and *Dionaea muscipula* [13]. Colchicine at long duration led to reduced survival ability because it is toxic to plants, especially tissue necrosis [14]. For growth performance, lowest root number and length, leaf number and length, fresh weight and dry weight were attained in tetraploid plants, while epidermal and stomatal cell length and width increased along with increased concentration and duration, and achieved largest size in tetraploid plants. Stomatal size is the most used indicator to analyze ploidy. Many studies have proved the exactness of stomata in identifying polyploidy in plants [15, 16]. The incompatible outcome between plant growth and epidermal or stomatal size yielded by tetraploid plants at high concentrations of colchicine concurred with previous study that growth performance of tetraploid *Spathoglottis eburnea* [17], *Rhynchostylis gigantean* [18] and Vanda hybrid [6] decreased, while stomatal size increased with lower stomatal density. Concentration and influence of colchicine on plants were dependent on incubation duration. Extended time of colchicine treatment inhibited growth by causing tissue damage and mitosis disturbance [19]. Plant growth reduction was caused by colchicine penetration into apical cell layers that impacted meristematic cell division [12]. Protocorms cultured at longer durations absorbed more colchicine that influenced cell division and spread throughout the cell, impairing cellular mechanisms and causing toxicity at high concentration and duration [20]. Moreover, medium supplementation by colchicine affected cytoplasm viscosity and led to aberration in cell functions [21]. However, some of our findings were inconsistent with a previous report. At 8 weeks of incubation duration, plantlets under 0.03% and 0.04% colchicine treatments were confirmed as polyploid, while the 0.05% treatment was not polyploid when checked by chromosome counting and flow cytometry analysis. Growth characteristics of the 0.05% colchicine treatment were greater than at 0.03% and 0.04%. This observation did not concur with a previous report that extended time of colchicine treatment inhibited growth by causing tissue damage and mitosis disturbance, relating to the random penetration of colchicine into cells and cytochimeras in treated plants. Meristematic tissues split into three histogenic layers as LI, LII, and LIII. Ploidy levels can be increased in all three layers or just one or two layers using antimitotic agents. Precise ploidy levels for the LI and LIII layers can be determined by guard cell investigations and chromosome counting, respectively [22]. Some tissues, organs or plantlets are not successful in polyploid induction, yielding large numbers of cytochimeras [23]. The 0.05% colchicine treatment plants at 8 weeks showed higher growth characteristics than the 0.03% and 0.04% treatments but their chromosome numbers remained diploid due to the occurrence of cytochimeras rather than polyploid. Direct methods such as chromosome counting and flow cytometry analysis have proved more effective and reliable to identify correct ploidy levels than physiological, morphological and anatomical traits as indirect methods.

Polyploidy in plants can be induced by soaking the protocorms in a liquid medium or using a solid colchicine medium. Our experiments showed that colchicine effectively induced polyploidy in a solid medium. This result concurred with Wu et al. [24] who demonstrated the success of solid media in inducing polyploidy in *Clematis heracleifolia*. Moreover, Dhooghe et al. [11] reported that solid medium colchicine usage effectively induced in vitro polyploidization in some plants including *Pyrus pyrifolia*, *Trifolium* sp., *Phlox subulata* and *Zingiber officinale*. However, the effect of colchicine on doubling chromosomes depended on various factors such as concentration, duration and treatment method, while different species had different responses to colchicine [25]. Moreover, one of the important benefits of tetraploid occurrence in plants is that they undergo physiological alteration that induces higher resistance to stress, including drought and water stress compared to diploids [11].

Growth performance and leaf surface anatomy data were further analyzed to investigate the response of *C. aloifolium* plantlets to different colchicine concentrations and culture periods. Results of Pearson’s correlation revealed positive correlations between the growth performance parameters, consistent with the PCA results (Figs. 7 and 8). All growth performance variables were in the upper right quadrant, where scores of treatments without colchicine were plotted. However, tetraploid plantlets from prolonged culture on 0.03% and 0.04% colchicine were plotted into the upper left quadrant and completely separated from diploids in other treatments. These results implied that growth performance parameters strongly declined when the number of chromosomes was doubled. By contrast, some studies reported improved growth in tetraploid plants. Tetraploid plants of *Citrus limon* showed larger leaves and higher plantlets than the diploids [16]. Several studies demonstrated that colchicine-induced polyploids ameliorated plant growth and development, while stunted growth in polyploid plants induced by colchicine was also reported. Octoploids of *Gladiolus grandifloras* provided more abnormal flowers, retarded growth and impaired vegetative developments [26], while decline in leaf and root characteristics were observed in *Rhynchostylis gigantea* var. *rubrum* orchids treated with various colchicine concentrations [18]. In this study, interrupted growth and development were also found in diploid plantlets treated with colchicine although not as severe as in the tetraploids. Negative effects occurring from treatments of colchicine resulted from its toxicity. Colchicine is commonly used to induce chromosome doubling due to its mitotic inhibition property that interrupts mitotic cell division and cytokinesis, leading to delayed growth and development [26]. Cellulose biosynthesis was also impacted, resulting in deficiency of cell wall components. Some genes involved with biosynthesis of plant metabolites and plant growth regulators were also downregulated due to colchicine [27]. These phenomena induce many abnormalities in morphology, physiology and anatomy of plants [23].

Positive correlations between width and length of stomatal and epidermal cell sizes were also observed. These were scattered in the same quadrant as the tetraploid plantlets but negatively correlated with stomatal and epidermal cell densities, indicating that the stomatal and epidermal cell sizes of *C. aloifolium* tetraploids were larger than the diploids, while stomatal and epidermal cell densities of the tetraploids decreased (Fig. 8). Tetraploids of *Jatropha curcas* provided significantly larger stomatal size with lower stomatal density [15]. Stomatal length and width increased in tetraploid *Citrus limon*, while stomatal density decreased [16]. Larger plant cell size is mainly caused by increase in chromosome copies, resulting in restricted cell division and reduction in the number of plant cells [23]. Positive correlations between growth performance and stomatal and epidermal cell densities were revealed. These parameters were indirectly correlated because growth performance variables of *C. aloifolium* plantlets responded to colchicine in the same way as stomatal and epidermal cell densities.

Response patterns of *C. aloifolium* plantlets to different colchicine concentrations and culture periods were clarified and explained using HCA and a heatmap. The analyzed variables were divided into two clusters. Growth performance and stomatal and epidermal cell densities highly responded in plantlets treated with low and medium colchicine concentrations or for short time culture periods, while plantlets treated with higher colchicine concentrations and prolonged culture periods, including tetraploids, showed larger stomatal and epidermal cell sizes (Fig. 9). Response patterns of plantlets treated with colchicine were clarified as five different patterns. Plantlets without treatments for two, four and eight weeks showed a similar pattern, with growth performance, stomatal and epidermal cell densities (cluster I) higher than stomatal and epidermal cell sizes (cluster II). After treatments at higher levels of colchicine for two, four and eight weeks the plantlets exhibited continuous decrease in expression levels of variables in cluster I, while expression levels in cluster II increased. Tetraploid plantlets treated with 0.03% and 0.04% colchicine for eight weeks demonstrated the largest stomatal and epidermal cell sizes but their growth was impacted and the densities of stomatal and epidermal cells decreased. Therefore, response levels of *C. aloifolium* plantlets to colchicine depended on concentration and culture period.

Interestingly, negative correlations were revealed between stomatal and epidermal cell densities and their sizes. Previous studies investigating these correlations revealed similar results. Inceer and Ozcan [28] reported that tetraploids of *Tripleurospermum* species possessed larger stomata and lower stomatal density than the diploids. All *Tripleurospermum* species were grouped into two clusters based on anatomical characteristics. Moreover, a negative correlation between density and cell size was also observed in barley [29], *Citrus limon* [16] and *Jatropha curcas* [15]. These results confirmed that anatomical differences can be used to identify polyploid and diploid plants, with the potential to increase the quality of orchids both as ornamental plants and as pharmaceutical raw materials.

## Conclusions

In vitro colchicine-mediated polyploid plants were successfully treated to induce tetraploid. *C. aloifolium* at 0.03% and 0.04% colchicine concentration for 8 weeks of incubation. The tetraploid plants obtained had lower rates of survival, response, root growth, leaf growth and fresh and dry weights compared to the diploids. Epidermal cell size and stomatal size were higher than in diploid plants but density was lower. Generally, the survival, response and growth performance decreased, while epidermal cell size and stomatal size increased at higher colchicine concentrations and incubation durations. Pearson’s correlation and PCA results showed correlations between growth performance and anatomical characteristics. Stomatal and epidermal cell sizes had strongly negative correlations with growth performance and densities. HCA results and the heatmap revealed and clarified the response patterns of *C. aloifolium* plantlets to colchicine, and showed that stomatal and epidermal cell sizes of tetraploid plantlets were larger than the diploids. Results suggested that unique autopolyploids generated here showed potential to increase the quality of orchids both as ornamental plants and as pharmaceutical raw materials.

## Methods

### Plant materials

*C. aloifolium* capsules were collected from Sawang Daen Din District, Sakon Nakhon Province, Thailand (17°27′24.1"N 103°27′20.2"E) and, identified by Dr. Pranee Nangngam and Dr. Anupan Kongbangkerd (Researchers at Department of Biology, Faculty of Science, Naresuan University, Phitsanulok, Thailand). A voucher specimen (number PNU05869) was deposited at the Department of Biology, Faculty of Science, Naresuan University, Phitsanulok, Thailand. Plants and seeds used in this study were permissible by the Plant Varieties Protection Office, Department of Agriculture, Thailand. For protocorm induction, capsules of *C. aloifolium* were sterilized in 5% (v/v) sodium hypochlorite (Clorox) with two drops of Tween 20 for 15 min and then dissected into two pieces. Mature seeds were transferred and cultured on solid New Dogashima Medium (NDM) [29] supplemented with 20 gl^−1^ sucrose, with pH adjusted to 5.4. The cultures were incubated at 25 ± 2 °C with a 16/8 h light/dark cycle (providing 40 μmol.m^−2^.s^−1^) for 12 weeks.

### Polyploid induction

Two-month-old *C. aloifolium* protocorms were used as explants. Protocorms were cultured on solid NDM supplemented with various concentrations of colchicine (0.00, 0.01, 0.02, 0.03, 0.04 and 0.05%) for 2, 4 and 8 weeks. The treated protocorms of the initial culture were transferred to solid NDM without colchicine. The cultures were incubated at 25 ± 2 °C with a 16/8 h light/dark cycle providing 40 μmol.m^−2^.s^−1^. The experiments comprised 18 treatments with 10 replicates and 10 protocorms per replicate.

### Polyploid analysis

Leaves of colchicine treated and control plantlets were used as explants for flow cytometry analysis. Relative DNA contents of each sample were analyzed using flow cytometry to identify plant polyploidy [17]. Leaves with 5 × 5 mm area were collected, placed in plastic Petri dishes, and then added with 0.4 ml of Quantum Stain NA UV 2 (A) as an extraction buffer solution. The plant material was then chopped with a sharp new razor blade for 10 s and incubated for 1 min before filtering through a disposable 50 μm mesh size filter into a small tube and added with 1.6 ml of Quantum Stain NA UV 2 (B) as staining reagent before incubating for 30 s. The nuclei sample was analyzed by Flow Cytometry (UV excitation) and results were represented as DNA histograms. Plantlets were classified as diploid, tetraploid or mixoploid based on the histogram peaks showing DNA content on the x-axis and nuclei number on the y-axis. Polyploidy levels of the plantlets were compared by DNA content between the control and treated plantlets [12].

### Chromosome counts

The root tips, measuring approximately 1–2 mm in length, were collected for chromosome counts in this study. The collected root tips were submerged in 1N HCl for 5 min, followed by warming at 60 °C on a slide warmer for 5–10 min. Subsequently, the root tips were washed three times with distilled water, stained with aceto-orcein on a glass slide, and covered with a cover glass, which was pressed slowly to ensure even cell scattering. The chromosomes were then observed and counted under a light microscope at magnifications of 400 × and 1000x.

### Stomatal observation

Mature leaves from the shoots of each treatment were used as plant organ materials. Epidermal peels were collected from the abaxial surfaces of the leaves. Each peel was washed with water and placed on a glass slide with a drop of 1% w/v safranin O solution in ethyl alcohol and then closed with a coverslip. All anatomical attributes were viewed and measured using a light compound microscope CH30 (Olympus) and a Zeiss Primo Star with MB2004 configuration AxioVision program. The length, width and density of stomata, epidermal length, width and density were measured in each treatment, comprising three slides with three areas on each slide of 1 mm^2^ each. Size measurements were obtained from three stomatal and three epidermal cells in each area, while stomatal and epidermal cell densities were calculated at each of the three sections on each slide.

### Growth performance

Survival percentage, response percentage, shoot number per protocorm (ptc), shoot length, root number per protocorm, root length, leaf number per protocorm, fresh weight and dry weight per replicate were evaluated. Survival percentage was calculated from protocorms showing a green color, while response percentage was calculated from protocorms that survived and produced roots and shoots in each replicate, following the method of Rohmah and Taratima [30].

$$\mathrm{Survival}\;\mathrm{percentage}=\left(\mathrm{Final}\;\mathrm{number}\;\mathrm{of}\;\mathrm{surviving}\;\mathrm{plants}/\mathrm{Initial}\;\mathrm{number}\;\mathrm{of}\;\mathrm{explants}\right)\times100$$

$$\mathrm{Response}\;\mathrm{percentage}=\left(\mathrm{Final}\;\mathrm{number}\;\mathrm{of}\;\mathrm{response}\;\mathrm{samples}/\mathrm{Initial}\;\mathrm{number}\;\mathrm{of}\;\mathrm{explants}\right)\times100$$

### Statistical analyses

All data were analyzed with SPSS by one-way analysis of variance (ANOVA). Differences between means were assessed by Duncan’s multiple range test (*p* ≤ 0.05). All data were presented as mean values with standard error (SE). Pearson’s correlation and principal component analysis (PCA) were used to identify the relationships between growth performance and leaf surface anatomical variables. The response pattern of *C. aloifolium* plantlets to colchicine concentrations and culture periods in colchicine containing media was studied using hierarchical cluster analysis (HCA). Pearson’s correlation, PCA and HCA were conducted using Origin 2022 software.

## Data Availability

The datasets used and/or analysed during the current study are available from the corresponding author on reasonable request.
